# Supplementary material for: Calcium-Bound S100P Protein Is a Promiscuous Binding Partner of the Four-Helical Cytokines
Source: Int J Mol Sci. 2022 Oct 9;23(19):12000. doi: 10.3390/ijms231912000 (PMC9569990; doi:10.3390/ijms231912000)
Supplement: Supplementary file 1 [file ijms-23-12000-s001.zip › ijms-1952407-supplementary.pdf]

Supplementary Materials

Calcium-Bound S100P Protein Is a Promiscuous Binding Partner of the Four-Helical Cytokines

Alexey S. Kazakov [1], Evgenia I. Deryusheva [1], Maria E. Permyakova [1], Andrey S. Sokolov [1], Victoria A. Rastrygina [1], Vladimir N. Uversky [1,2,*], Eugene A. Permyakov [1] and Sergei E. Permyakov [1,*]

[1] Institute for Biological Instrumentation, Pushchino Scientific Center for Biological Research of the Russian Academy of Sciences, Institutskaya str., 7, Pushchino, Moscow Region 142290, Russia

[2] Department of Molecular Medicine and USF Health Byrd Alzheimer's Research Institute, Morsani College of Medicine, University of South Florida, Tampa, FL 33612, USA

* Correspondence: vuversky@usf.edu (V.N.U.); permyakov.s@gmail.com (S.E.P.);
Tel.: +7-(495)-143-7740 (S.E.P.); Fax: +7-(4967)-33-05-22 (S.E.P.)

Table S1. The four-helical cytokine samples that did not reveal specificity to S100P, according to SPR spectroscopy (cytokine immobilization on the sensor chip surface by amine coupling).

Full name	*Abbreviation*	*UniProt ID*	*Manufacturer*	*Cat. number*	*Source*
Short-chain cytokines					
Macrophage colony-stimulating factor 1	M-CSF	P09603	PeproTech	300-25	*E.coli*
Interleukin-2	IL-2	P60568	PeproTech	AF-200-02	*E.coli*
Interleukin-4	IL-4	P05112	PeproTech	AF-200-04	*E.coli*
Long-chain cytokines					
Interleukin-7	IL-7	P13232	SCI-Store (Russia)	PSG240-10	CHO
Thymic stromal lymphopoietin	TSLP	Q969D9	PeproTech	300-62	*E.coli*
Chorionic somatomammotropin hormone 1	PL	P0DML2	R&D Systems	5757-PL/CF	CHO
Interleukin-6#	IL-6	P05231	PeproTech	200-06	*E.coli*
Leukemia inhibitory factor#	LIF	P15018	PeproTech	300-05	*E.coli*
Interleukin-12	IL-12	P29459* & P29460	PeproTech	200-12H	HEK293
Interleukin-23	IL-23	Q9NPF7* & P29460	PeproTech	200-23	Hi-5
Interferons/IL-10					
Interferon α-2	IFN-α2	P01563	Vector-Medica (Russia)	n/a	*E.coli*

Interferon γ	IFN-γ	P01579	Pharmaclon (Russia)	n/a	*E.coli*

[#] ref. [1]

* denotes the chain used for SCOP 2 [2] family assignment

n/a, not applicable

Table S2. The concentrations of four-helical cytokines under normal and pathological conditions.

<table>
<tr><th rowspan="3">Cytokine</th><th rowspan="3">Condition</th><th rowspan="3">Liquid</th><th colspan="4">Detected level</th><th rowspan="3">Reference</th></tr>
<tr><th colspan="2">Disease</th><th colspan="2">Healthy control</th></tr>
<tr><th>pg/ml</th><th>pM</th><th>pg/ml</th><th>pM</th></tr>
<tr><td colspan="8">Short-chain cytokines</td></tr>
<tr><td rowspan="8">GM-CSF</td><td>Axial spondyloarthritis</td><td>Serum</td><td>150</td><td>10.36</td><td>62</td><td>4.28</td><td>[3]</td></tr>
<tr><td>Systemic sclerosis</td><td>Serum</td><td>120.9±125.5</td><td>8.29±8.67</td><td>20.1±12.3</td><td>1.39±0.85</td><td>[4]</td></tr>
<tr><td>Chronic myelogenous leukemia</td><td>Serum</td><td>3.9-55</td><td>0.27-3.8</td><td>-</td><td>-</td><td>[5]</td></tr>
<tr><td>Rheumatoid arthritis</td><td>Plasma</td><td>366±61 (severe)
376±44 (moderate)</td><td>25.27±4.22 (severe)
25.97±3.04 (moderate)</td><td rowspan="3">174 ± 18</td><td rowspan="3">12.02±1.24</td><td rowspan="3">[6]</td></tr>
<tr><td>Systemic lupus erythematosus</td><td>Plasma</td><td>256±41</td><td>17.67±2.83</td></tr>
<tr><td>Spondyloarthropathy</td><td>Plasma</td><td>190±32</td><td>13.02±2.21</td></tr>
<tr><td>Relapsing-remitting multiple sclerosis</td><td>Serum</td><td>27.8±18.4 (relapse)
19.4±13.8 (remission)</td><td>1.92±1.27 (relapse)
1.34±0.95 (remission)</td><td>17.9±13.3</td><td>1.24±0.91</td><td>[7]</td></tr>
<tr><td>Healthy</td><td>Serum</td><td>-</td><td>-</td><td>38.3
(26.3–63.8)</td><td>2.64
(1.82-4.41)</td><td>[8]</td></tr>
<tr><td rowspan="3">IL-3</td><td>Homozygous beta-thalassemia (children)</td><td>Plasma</td><td>6.9±17.1</td><td>0.46±1.13</td><td>0.2±0.5</td><td>0.013±0.033</td><td>[9]</td></tr>
<tr><td>Healthy</td><td>Serum</td><td>-</td><td>-</td><td>< 12</td><td>< 0.8</td><td>[8]</td></tr>
<tr><td>Healthy</td><td>Serum</td><td>-</td><td>-</td><td>5.8 men
8.0 women</td><td>0.38 men</td><td>[10]</td></tr>
</table>

						0.53 women	
	Chronic schizophrenia	Serum	27.0±19.7	1.79±1.31	14.6±10.4	0.97±0.69	[11]
IL-5	Healthy	Serum	-	-	< 3.1	< 0.2	[8]
	Hepatocellular carcinoma	Serum	10.97 (0.29–17042.32)	0.83 (0.02-1.30)	-	-	[12]
	Acute asthmatic attack	Serum	4.84±1.68	0.37±0.13	0.32±0.26	0.024±0.020	[13]
	Asthma	Serum	9.5 (atopic) 8.1 (non-atopic)	0.72 (atopic) 0.62 (non-atopic)	4.4	0.33	[14]
		Serum	0–46.75	0-3.56	0–5.20	0-0.40	[15]
	Chronic obstructive pulmonary disease	Serum	6.4±3.2	0.49±0.24	-	-	[16]
IL-9	Hodgkin's lymphoma	Serum	up to 3350	up to 237	-	-	[17]
	Healthy	Serum	-	-	113.6 men 573.3 women	8.1 men 40.6 women	[10]
	Rheumatoid arthritis	Serum	4.77±3.618	0.34±0.26	1.22±0.706	0.09±0.05	[18]
	Systemic lupus erythematosus	Serum	12.26±25.235	0.87±1.79			
	Colon cancer	Plasma	1.29	0.09	2.53	0.18	[19]
	Inflammatory bowel disease	Serum	40±5	2.83±0.35	< 6	< 0.4	[20]
	Diffuse large B-cell lymphoma	Serum	1.43±0.64	0.10±0.05	0.82±0.15	0.06±0.01	[21]
	Allergic rhinitis	Serum	12.6 (5.03–24.11) (exposed to allergen)	0.9 (0.3-1.7)	-	-	[22]
			4.83	0.3			

			(1.97–14.01)<br>(not exposed to allergen)	(0.1-1.0)			
	Systemic sclerosis	Serum	84.6±76.0	5.99±5.39	40.4±41.7	2.86±2.95	[23]
	Systemic lupus erythematosus		50.7±52.0	3.59±3.68			
	Dermatomyositis		50.6±55.8	3.58±3.95			
	Atopic dermatitis		41.8±38.8	2.96±2.75			
IL-13	Asthma	Serum	0–157.10	0–9.93	0–5.40	0–0.38	[15]
	Healthy	Serum	-	-	11.2 men<br>14.1 women	0.8 men<br>1.0 women	[10]
	Insulin resistance	Serum	37.69±17.82	2.38±1.13	15.88±6.71	1.13±0.47	[24]
	Chronic rhinosinusitis with nasal polyps	Serum	0.98±1.56	0.06±0.10	0.34±0.16	0.024±0.011	[25]
	Acute asthmatic attack	Serum	50.85±27.30	3.22±1.73	8.10±5.40	0.57±0.38	[13]
	Ocular Behcet's disease	Serum	38.7±59.6<br>(active ocular BD)<br>9.21±2.03<br>(ocular BD in remission)<br>10.3±1.65<br>(nonocular BD in remission)	2.45±3.77<br>0.58±0.13<br>0.65±0.10	7.83±1.85	0.56±0.13	[26]
	Hepatocellular carcinoma	Serum	38.425<br>(7.73–539.93)	2.43<br>(0.49-34.14)	-	-	[12]
	Psoriatic arthritis	Serum	2<br>(2–2)	0.13<br>(0.13-0.13)	2 (2–6.2)	0.14 (0.14-0.44)	[27]

	Rheumatoid arthritis		10.2 (2–28.3)	0.64 (0.13-1.79)			
	Osteoarthritis		2 (2–5.8)	0.13 (0.13-0.37)			
	Systemic sclerosis	Serum	0.84±0.65	0.05±0.04	0.35±0.18	0.025±0.012	[28]
IL-15	Polymyositis/dermatomyositis	Serum	47.6±170 (active)	3.73±13.31	28.5±28.89	2.5±2.3	[29]
			25.15±240 (inactive)	1.97±18.79			
	Healthy	Serum	-	-	< 2.1	< 0.16	[8]
	Essential hypertension	Serum	88.7±18.7 (severe organ damage) 55.2±6.9 (mild organ damage) 51.0±8.3 (no organ damage)	6.94±1.46 4.32±0.54 3.99±0.65	-	-	[30]
	Kawasaki disease	Serum	11.5±5.8 (acute stage) 1.3±0.9 (subacute stage)	0.91±0.45 0.10±0.07	0.9 (1.0)	0.07 (0.08)	[31]
	Prostate cancer	Serum	208.07±48.50 (early stage prostate cancer) 50.51±14.34 (benign prostatic hyperplasia)	16.28±3.79 3.95±1.12	-	-	[32]

	Multiple sclerosis	Serum	4.2±1.36<br>5.56±3.6 (PPMS)<br>4.13±0.61 (RRMS)<br>4.26±0.73 (SPMS)<br>4.13±0.45 (IDS)	0.33±0.10<br>0.44±0.28<br>0.33±0.05<br>0.34±0.06<br>0.33±0.04	-	-	[33]
	Inflammatory neurological diseases		1.95±0.41	0.16±0.03			
	Non-inflammatory neurological diseases		2.96±1.13	0.23±0.09			
	Ulcerative colitis (moderate to severe)	Serum	0-490	0-38	-	-	[34]
	Hepatocellular carcinoma	Serum	77.4±78	6.06±6.11	-	-	
	Bacteremic melioidosis	Serum	49.4 (12.4-338.8)	3.87 (0.98-26.52)	12.8 (< 8.2-122.2)	1.00 (< 0.64-9.57)	[35]
	Non-bacteremic melioidosis	Serum	31.3 (11.6-2743)	2.45 (0.91-214.74)			
IL-21	Psoriasis	Serum	68.86±32.13	4.31±2.01	50.04±23.56	3.13±1.47	[36]
	Systemic lupus erythematosus	Serum	24.64 (21.15–28.46)	1.54 (1.32-1.78)	27.74 (23.28–34.64)	1.73 (1.46-2.17)	[37]
	Atopic dermatitis	Serum	454 (up to 6 293)	28.39 (up to 394)	50.6	3.16	[38]
	Chronic hepatitis B	Serum	303.54±152.77	18.98±9.55	68.24±9.06	4.27±0.57	[39]

	Coronary artery disease	Serum	187.0±6.0	11.7±0.4	120.5±5.0	7.5±0.3	[40]
THPO	Cirrhosis	Serum	69±12	1.82±0.32	49±9	1.3±0.24	[41]
	Myelosuppression after intensification chemotherapy of acute leukemia in complete remission	Serum	-	18.46±9.70	-	0.84±0.40	[42]
	Aplastic anemia		-	16.03±9.44			
	Acute lymphoblastic leukemia		-	10.36±5.57			
	Malignant lymphoma		-	2.79±2.27			
	Multiple myeloma		-	3.34±0.20			
	Chronic lymphocytic leukemia		-	1.71±3.91			
	Myeloproliferative disorders		-	1.99±1.47			
	Acute myelogenous leukemia		-	2.27±1.25			
	Hypoplastic leukemia		-	2.76±2.23			
	Myelodysplastic syndrome		-	0.42±0.60			
	Liver cirrhosis		-	1.50±0.92			
	Idiopathic thrombocytopenic purpura		-	2.08±1.41			
	Thrombocytopenia	Serum	>500	>14.1	<100	<2.8	[43]
	Ovarian cancer	Serum	114 (25–435)	3.2 (0.7-12.3)	-	-	[44]
	Benign ovarian disease		74 (34–189)	2.1 (1.0-5.3)			
	Consumptive thrombocytopenia	Serum	63 (48–98)	1.8 (1.4-2.8)	7–99	0.2-2.8	[45]

	Hypoproliferative thrombocytopenia		706 (358–1546)	19.9 (10.1-43.6)			
Long-chain cytokines							
IL-31	Paediatric atopic dermatitis	Serum	1600 (1457.8±770.4) (flare) 1040 (958.7±419.5) (quiescence)	101 (92±49) 66 (61±26)	220 (197.3±91.9)	14 (12±6)	[46]
	Osteoporosis	Serum	43.12±6.97	2.72±0.44	29.58±6.09	1.86±0.38	[47]
	Early axial spondyloarthritis	Serum	12.6±15.4	0.79±0.98	1.8±4.0	0.11±0.25	[48]
	Chronic kidney disease-associated pruritus	Serum	679.9±1112.3	42.9±70.2	57.3±65.1	3.6±4.1	[49]
	Atopic dermatitis	Serum	up to 10 000	up to 631	up to 1 200	up to 76	[50]
	Endometrial cancer	Serum	165.80±39.03 (94.43–240.65) (before operation) 128.91±29.48 (53.54–187.54) (after operation)	10.47±2.46 (5.97-15.20) 8.14±1.86 (3.38-11.84)	77.24±25.85 (32.50–142.82)	4.87±1.63 (2.05-9.02)	[51]
IL-27	Systemic sclerosis	Serum	302.8 (101.6-1034.4) 385 (109-826.3)	6.33 (2.12-21.62) 8.05 (2.28-17.27)	104.2 (51-184.2)	2.18 (1.06-3.85)	[52]
	Chronic HIV-1 Infection	Serum	2 008.0±274.8	42.0±5.7			[53]

			(<50 copies/ml group)<br>1 468.7±172.3<br>(51–10 000 copies group)<br>1 237.9±127.3<br>(10 001–100 000 copies/ml group)<br>1 590.1±223.7<br>(>100 000 copies/ml group)	30.7±3.6<br>25.9±2.7<br>33.2±4.7	2 990.7±682.1	62.5±14.3	
	Childhood immune thrombocytopenia	Serum	770.6	16.1	373.8	7.8	[54]
	Ocular Behcet's disease	Serum	320±57.5<br>(active ocular BD)<br>316±47.3<br>(ocular BD in remission)<br>305±32.4<br>(nonocular BD in remission)	6.69±1.20<br>6.60±0.98<br>6.38±0.67	347±50.1	7.25±1.05	[26]
	Ischemic heart disease	Serum	38.00±14.38<br>(acute myocardial infarction)<br>35.77±18.93<br>(unstable angina)	0.80±0.30<br>0.75±0.40	24.91±14.96	0.52±0.31	[55]

	Diabetic retinopathy	Serum	240.900<br>(42.224–617.810)	5.0<br>(0.9-12.9)	2 712.310<br>(1 005.375–5 786.877)	56.68<br>(21-120)	[56]
	Type 1 diabetes mellitus	Serum	22.06	0.46	14.5	0.30	[57]
	Type 1 diabetes mellitus + Hashimoto's disease		27.82	0.59			
	Healthy	Serum	-	-	Most 100-1 000<br>Minority >2 000<br>4.4% > 6 000	Most 2.1-20.9<br>Minority > 41.8<br>4.4% > 125.4	[58]
IL-35	Pre-eclampsia	Serum	729±335	15.90±7.31	483.9±242	10.55±5.28	[59]
	Gastric cancer	Serum	17.559±13.266	0.38±0.29	8.077±3.801	0.18±0.08	[60]
	Prostate cancer	Serum	20.01±7.03	0.44±0.15	11.60±2.49	0.25±0.05	[61]
	Rheumatoid arthritis	Serum	12.9<br>(6.4-16.9)	0.28<br>(0.14-0.37)	6.7<br>(4.3-12.2)	0.15<br>(0.09-0.27)	[62]
	Multiple sclerosis	Serum	49.3±3.7	1.08±0.08	69.5±7.8	1.52±0.17	[63]
	Systemic sclerosis	Serum	83.9<br>(45.1–146.1)	1.83<br>(0.98-3.19)	36.2<br>(17.2–49.4)	0.79<br>(0.38-1.08)	[64]
	Systemic sclerosis	Serum	5.08±0.76<br>7.75±1.36<br>(fibrosis)<br>3.08±0.70<br>(without fibrosis)	0.111±0.017<br>0.169±0.030<br>(fibrosis)<br>0.067±0.015<br>(without fibrosis)	1.89±0.69	0.041±0.015	[65]
G-CSF	Healthy	Serum	-	-	45.5	2.4	[8]

					(34–53.6)	(1.8-2.80)	
	Healthy	Serum	-	-	70.3 men<br>93.7 women	3.7 men<br>4.9 women	[10]
	Acute stage of infection	Serum	731.8±895.0<br>(30-3 199)	38.6±47.1<br>(1.6-168.5)	25.3±19.7<br>< 100 in all cases	1.33±1.03<br>< 5.27 in all cases	[66]
	Breast cancer	Serum	92.81±594.54	4.88±31.31	0.00	0.00	[67]
	Acute bacterial infection	Serum	799±1 501	42±79	-	-	[68]
	Viral infection		58±34	3.1±1.8			
	Atypical pneumonia		60±33	3.2±1.7			
	Chronic myelogenous leukemia	Serum	150-2 830	7.9-149.1	-	-	[5]
	Urothelial carcinoma	Serum	147	7.74	< 37	1.95	[69]
	Various disorders	Serum	46 to > 2 000	2.4 to >105.3	< 30 to 163	<1.6 to 8.6	[70]
	Septic shock after surgery	Serum	up to 20 000	up to 1050	<100	< 5.27	[71]
GH	Gastric cancer	Serum	3 140±3 120<br>(200-10 900)	142±141<br>(9-493)	690±1 600<br>(100-9 000)	31±72<br>(4.5-406.7)	[72]
	Colorectal cancer		3 010±2 910<br>(200-9 900)	136±132<br>(9-447)			
	Healthy	Serum	-	-	400-10 000<br>(adult males)<br>1 000-14 000<br>(adult females)<br>10 000-50 000<br>(children)	18-452<br>45-633<br>452-2 260	[73]
	Acromegaly	Serum	≥ 1 000	≥45	<1 000	< 45	[74]

	Healthy	Serum	-	-	2 400±2 800 (boys) 2 500±3 100 (girls)	108±127 113±140	[75]
GH-V	Down syndrome pregnancy	Maternal serum	-	-	531 (8th week) 935 (9th week) 962 (10th week) 1 103 (11th week) 1 204 (12th week) 1 169 (13th week)	23.79 (8th week) 41.89 (9th week) 43.10 (10th week) 49.42 (11th week) 53.94 (12th week) 52.37 (13th week)	[76]
	Pre-eclampsia	Maternal serum	23 076 (3 473-94 256)	1 030 (156-4 220)	12 157 (2 617-34 016)	545 (117-1 520)	[77]
PRL	Healthy	Serum	-	-	< 20 000 (men) < 25 000 (nonpregnant women) 80 000-40 0000 (pregnant women)	< 870 (men) < 1 090 (nonpregnant women) 3 490-17 470 (pregnant women)	[27]
	Phenylketonuria	Serum	12 000 (3 000–75 000)	524 (131-3 280)	1 400–24 000 (females) 1 600–10 700	61-1 050 (females) 70-467 (males)	[78]

					(males)		
	Systemic lupus erythematosus	Serum	17 400±15 100	760±660	6 300±3 200	275±140	[79]
	Rheumatic autoimmune diseases		13 100±10 300	572±450			
	Psoriasis	Serum	11 290±8 050 (3 800-39 600)	493±352 (166-1 730)	7 900±3 930 (2 800-18 600)	345±172 (122-812)	[80]
	Breast cancer	Serum	5 000–77 900	218-3 400	<20 000	<873	[81]
	Type 2 diabetes mellitus	Serum	36 840±6 290	1 610±275	10 660±2 450	466±107	[82]
LEP	Asthma	Serum	13 810±10 560	862±659	6 320±5 200	394±324	[83]
	Multiple sclerosis	Serum	-	50-6250	-	-	[84]
	Multiple sclerosis	Serum	11 135±6 453 (RRMS)<br>30 258±14 435 (SPMS)<br>30 301±12 709 (PPMS)	695±403<br>1 890±901<br>1 890±793	-	-	[85]
	Non–small cell lung cancer	Serum	8 500±1 600<br>10 700±2 500 (without weight loss)<br>7 200±2 100 (weight loss)	530±100<br>668±156<br>449±131	12 800±2 400	799±150	[86]
	Obesity	Serum	44 920±26 490 (women)<br>15 150±9 500 (men)	2 800±1 650 (women)<br>945±593 (men)	21 810±17 110 (women)<br>7310±5080 (men)	1 360±1 070 (women)<br>456±317 (men)	[87]
	Obesity	Serum	11 390±8 790 (boys)<br>12 640±9 330 (girls)	711±549 (boys)<br>789±582 (girls)	4 800±5 250 (boys)<br>4 810±4 430 (girls)	300±328 (boys)<br>300±276 (girls)	[88]

Interferons/IL-10							
IL-10	Healthy	Serum	-	-	12.6 (8.5–16.7)	0.68 (0.46-0.90)	[8]
	Healthy	Serum	-	-	2.7	0.14	[10]
	Melanoma	Serum	15–480	0.80-25.74	<3.0	<0.16	[89]
			8.75	0.47	<3.0	<0.16	
	Gastric cancer		27.52	1.48	<12	<0.64	
			>21.0	>1.13	<3.0	<0.16	
	Pancreatic cancer		>10.0	>0.54	N/D	<0.16	
			>9.8	>0.53	3.0	0.16	
	Colorectal cancer		97.36	5.22	24.53	1.32	
			16.09	0.86	5.1	0.27	
	Hepatic cancer		12	0.64	6.3	0.34	
	Hodgkin lymphoma		>10	>0.54	7.1	0.38	
	Non-Hodgkin lymphoma		>7.98	>0.43	<5.0	<0.28	
	Lung cancer		>38.16	>2.05	32.55	1.75	
			21.4	1.15	9.2	0.49	
	B-cell lymphoma		26.0	1.39	18.0	0.97	
	Chronic lymphocytic leukemia		74	3.97	<13.68	<0.73	
	Psoriasis	Serum	89.5±18.7	4.8±1	117.2±23.4	6.29±1.25	[90]
IL-20	Rheumatoid arthritis	Serum	30.2 (19.1–58.5)	1.72 (1.09-3.34)	13.1 (11–15.1)	0.75 (0.63-0.86)	[91]
	Psoriasis	Serum	20.418±20.587	1.16±1.17	2.703±3.043	0.15±0.17	[92]
	Non–small cell lung cancer	Serum	40.35 (29.86-63.81)	2.30 (1.70-3.64)	37.73 (32.49-50.80)	2.15 (1.85-2.90)	[93]
	Rheumatoid arthritis	Plasma	282 (134–438)	16.09	-	-	[94]

				(7.65-24.99)			
	Osteoarthritis		124 (110–147)	7.07 (6.28-8.39)			
	Rheumatoid arthritis	Plasma	89 (45–643)	5.08 (2.57-36.69)	71 (40–80)	4.05 (2.28-4.56)	[95]
	Systemic sclerosis	Serum	56.89±11.6	3.25±0.66	79.11±18.2	4.51±1.04	[96]
	Primary Raynaud's phenomenon		71.42±11.8	4.07±0.67			
IL-22	Psoriasis	Serum	71.600±150.759	4.27±9.00	2.358±2.486	0.14±0.15	[92]
	Non–small cell lung cancer	Serum	10.66 (1.44-70.34)	0.64 (0.09-4.20)	4.69 (0.35-12.29)	0.28 (0.02-0.73)	[93]
	Systemic sclerosis	Serum	68.3±44.4	4.09±2.65	59.52±16.7	3.55±1.00	[96]
	Primary Raynaud's phenomenon		63.1±11.8	3.77±0.70			
	Epithelial ovarian cancer	Plasma	34.19±4.58	2.04±0.27	22.1±1.71	1.32±0.10	[97]
	Benign ovarian epithelial neoplasm		23.1±1.78	1.38±0.11			
	Hepatocellular carcinoma	Serum	299.675 (5.99–1963.32)	17.89 (0.36-117.2)	-	-	[12]
	Multiple myeloma	Serum	75.5±63.3	4.51±3.78	8.1±5.7	0.48±0.34	[98]
	Low-grade squamous intraepithelial lesion	Serum	168.2	10.0	36.91	2.20	[99]
	High-grade squamous intraepithelial lesion		61.48	3.67			
	Breast cancer	Serum	317.53±14.33	18.96±0.86	62.67±19.11	3.74±1.14	[100]

	Type 2 autoimmune hepatitis	Serum	55.26	3.3	0.1	0.006	[101]
IL-24	Rheumatoid arthritis	Plasma	2 210 (310–7290)	122 (17-401)	80 (0–3630)	4.4 (0-200)	[94]
	Spondyloarthritis		2 020 (0–6830)	111 (0-376)			
	Breast cancer	Serum	160.65±55	8.84±3.03	27.4±8.5	1.51±0.47	[102]
	Gastric cancer + H. Pylori infection		76.2±16.27	4.2±0.9			
	Gastric cancer		72.5±17.84	3.99±0.98			
	H. Pylori infection		32.78±12.96	1.8±0.71			
	Systemic lupus erythematosus	Serum	1 375.70 (812.92–2 096.54)	75.76 (44.76-115.45)	334.70 (129.13–634.91)	18.43 (7.11-34.96)	[103]
IL-26	Systemic lupus erythematosus	Serum	4 040±11 660	230±663	740±2 020	42±115	[104]
	Neurosyphilis	Serum	6870	391	1670	95	[105]
	Severe asthma	Serum	1 100±390	63±22	550±250	31±14	[106]
	Behçet's disease	Serum	4 800±1 320 (active) 2 770±1 026 (inactive)	273±75 158±58	310±140	18±8	[107]
	Rheumatoid arthritis	Serum	2 430±3 800	138±216	30±40	1.71±2.27	[108]
	Other inflammatory arthritis	Serum	8 820±16 320	502±928	30±40	1.71±2.27	
IFN-ω1	Dermatomyositis	Serum	0-120	0-5.95	0-60	0-2.98	[109]

Table S3. The structures (PDB [110] entries or AlphaFold2 [111] predictions) of the human cytokines used for structural modelling of their complexes with Ca^{2+}-loaded human S100P dimer (chains B, D of PDB entry 2MJW) using ClusPro docking server [112].

Cytokine	*PDB entry*	*Chain*	*Method*	*Comments*
Short-chain cytokines				
GM-CSF	1CSG	A	X-ray	
IL-3	6NMY	I	X-ray	W6Y substitution; complex with the receptor chains
IL-5	1HUL	A	X-ray	dimeric structure
IL-9	-	-	AlphaFold2	
IL-13	1GA3	A	NMR	
IL-15	4GS7	A	X-ray	complex with the receptor chains
IL-21	2OQP	A	NMR	
THPO	1V7M	V	X-ray	complex with a neutralizing antibody fragment; only structured fragment of the protein 22-184 was used for docking
Long-chain cytokines				
IL-31	-	-	AlphaFold2	
G-CSF	1GNC	A	NMR	
GH	-	-	AlphaFold2	
GH-V	-	-	AlphaFold2	
PRL	1RW5	A	NMR	
LEP	1AX8	A	X-ray	W100A substitution
Interferons/IL-10				
IL-10	2ILK	A	X-ray	

IL-20	4DOH	A	X-ray	complex with the receptor chains
IL-22	1M4R	A	X-ray	
IL-24	6DF3	C	X-ray	N34Q, N48Q, Y73H, N75Q substitutions; complex with the receptor chains
IL-26	-	-	AlphaFold2	
IFN-ω1	3SE4	B	X-ray	N83Q substitution; complex with the receptor chains

Table S4. The residues of the binding sites for the S100P-cytokine complexes predicted using ClusPro docking server [112]. The contact residues included into 5 or more docking models were considered as the most probable residues of the binding site. The numbering of the residues is according to the PDB entries. The residues of receptor-binding sites of the cytokines are shown in bold. The following receptors are taken into account: GM-CSF receptor subunit α (PDB entry 4RS1), IL-3 receptor subunit α (PDB ID: 5UV8), IL-5 receptor subunit α (PDB ID: 3VA2), IL-15 receptor subunits α, β and γ (PDB ID: 4GS7), IL-21 receptor (PDB ID: 3TGX), G-CSF receptor (PDB ID: 2D9Q), GH receptor (PDB ID: 3HHR), IL-20 receptor subunits α and β (PDB ID: 4DOH), IFN-α/β receptors 1 and 2 (PDB ID: 3SE4).

<table>
<tr><td colspan="3">Short-chain cytokines</td></tr>
<tr><td colspan="2">S100P dimer</td><td rowspan="2">GM-CSF</td></tr>
<tr><td>Chain B</td><td>Chain D</td></tr>
<tr><td>P42,G43,F44,Y88,F89</td><td>M1,T2,E5,G9,D13,R17</td><td>Q11,W13,E14,V16,N17,Q20,E21,R23,R24,L28,K72,L115,F119</td></tr>
<tr><td colspan="2">S100P dimer</td><td rowspan="2">IL-3</td></tr>
<tr><td>Chain B</td><td>Chain D</td></tr>
<tr><td>G43,Y88,F89,A92</td><td>M1,E5</td><td>Y13,S17,R43,L82,P83,C84,L85,P86,L87,A88,T89,N120</td></tr>
<tr><td colspan="2">S100P dimer</td><td rowspan="2">IL-5</td></tr>
<tr><td>Chain B</td><td>Chain D</td></tr>
<tr><td>E40, P42,G43,C85,Y88,F89,A92</td><td>M1,T2,E5</td><td>T19,L20,A23,N24,Q54,V56,Q57</td></tr>
<tr><td colspan="2">S100P dimer</td><td rowspan="2">IL-9</td></tr>
<tr><td>Chain B</td><td>Chain D</td></tr>
<tr><td>E3,C85,Y88,F89</td><td>M1,E5,G9,I12,D13,S16,R17</td><td>C3,Q115,K118,M122,R123,K125,I126</td></tr>
<tr><td colspan="2">S100P dimer</td><td rowspan="2">IL-13</td></tr>
<tr><td>Chain B</td><td>Chain D</td></tr>
<tr><td>G43,F44,C85,F89</td><td>E5</td><td>P15,S16,T17,R74,G78,S90,N126</td></tr>
<tr><td colspan="2">S100P dimer</td><td rowspan="2">IL-15</td></tr>
<tr><td>Chain B</td><td>Chain D</td></tr>
</table>

M1,E5,P42,F44,Y88,F89	M1,T2,E5,F44,Y88,F89,A92	N1,W2,V3,K10,**D30**,**H32**,P33,K36,S76,N77,N79,V80,T81,H105,**Q108**,M109,**I111**,**N112**,T113,S114
**S100P dimer**		**IL-21**
Chain B	Chain D	
M1,T2,E3,E5,T6,D13,R17,E40,Y88,G93	M1,T2,E3,E5,T6,M10,D13,S16,R17,E40,F89	M5,Q6,G7,Q8,D9,**R10**,H11,**R14**,N46,W51,**R81**,**P84**,**S85**,R90,R91,H94,R95,H127,L128,S129,R131
**S100P dimer**		**THPO**
Chain B	Chain D	
E3,R17,Y88,F89,A92,G93,L94	M1,E5,D13,S16,R17,T25,Q26	S7,R11,H14,H17,V38,F40,S41,L42,H115,L123,R130,R134,F135,L138
Long-chain cytokines		
**S100P dimer**		**IL-31**
Chain B	Chain D	
F44,Q46,C85,Y88,F89	E5	S1,H2,L4,P5,V6,R7,L9,R10,P11,D53,Q55,P56,N58,F93,Q94,D95
**S100P dimer**		**G-CSF**
Chain B	Chain D	
G43,F44,C85,Y88,F89,A92	M1,E5,I12,G43,F44,F89	Q71,L72,A73,**Q120**,Q121,E123,**E124**,L169
**S100P dimer**		**GH**
Chain B	Chain D	
M1,E5,G43,F44,C85,Y88,F89	M1,E5,P42,G43,Y88,F89	D11,**L15**,**H18**,**Q22**,**R64**,Q68,**R178**,I179,C182,R183,E184,G187,S188,**C189**,**G190**,F191
**S100P dimer**		**GH-V**
Chain B	Chain D	
D13,F44,Y88	F44	F1,P2,T3,I4,R8,N12,R16,R112,D116,E119,Q122,T123,W126,R127,D130,Q137
**S100P dimer**		**PRL**
Chain B	Chain D	
L41,F44,I81,C85,Y88,F89	E5	L1,I3,C4,G6,G7,A9,R10,C11,Q12,V13,T14,R16,D17,D20,R21,R125,E128,K190,I194,H195

S100P dimer		LEP
Chain B	Chain D	
C85,Y88,F89	M1,E5,D13	V1,I3,D85,H88,F92,S93,K94,S95,C96,H97,L98,C146
	Interferons/IL-10	
**S100P dimer**		**IL-10**
Chain B	Chain D	
E5,Y88,F89,G93,L94	D13,Y88,F89	Q70,K117,S118,A120,V121,K125,M140,F143,F146,Y149,I150,Y153,M154,I158
**S100P dimer**		**IL-20**
Chain B	Chain D	
E5,M8,Y88,F89	E5,F44,C85,Y88,F89	S9,V11,R60,R64,**K97**,D98,R100,H103,A104,H105,M106,T107,C108,H109,C110
**S100P dimer**		**IL-22**
Chain B	Chain D	
P42,G43,F44,C85,Y88,F89	M1,T2,E5	H6,F47,H48,G49,M52,S53,E54,R55,D104,N143,A144,I146
**S100P dimer**		**IL-24**
Chain B	Chain D	
F44,I81,C85,Y88,F89	M1,T2,E5,D13	Y56,H59,T60,P102,Q104,N106,F109,S110,R112,D113,H116,R117,L120,R124,K127,Q128
**S100P dimer**		**IL-26**
Chain B	Chain D	
P42,G43,C85,Y88,F89	M1,T2,E5,G9,D13	Y12,S102
**S100P dimer**		**IFN-ω1**
Chain B	Chain D	
E5,M8,Y88,F89	E5,G43,F44,C85,Y88,F89	**R14**,**H21**,Q22,R24,R25,I26,S27,**P28**,**F29**,L30,K33,**R35**,D140,R147,**M148**,**M151**,**F155**

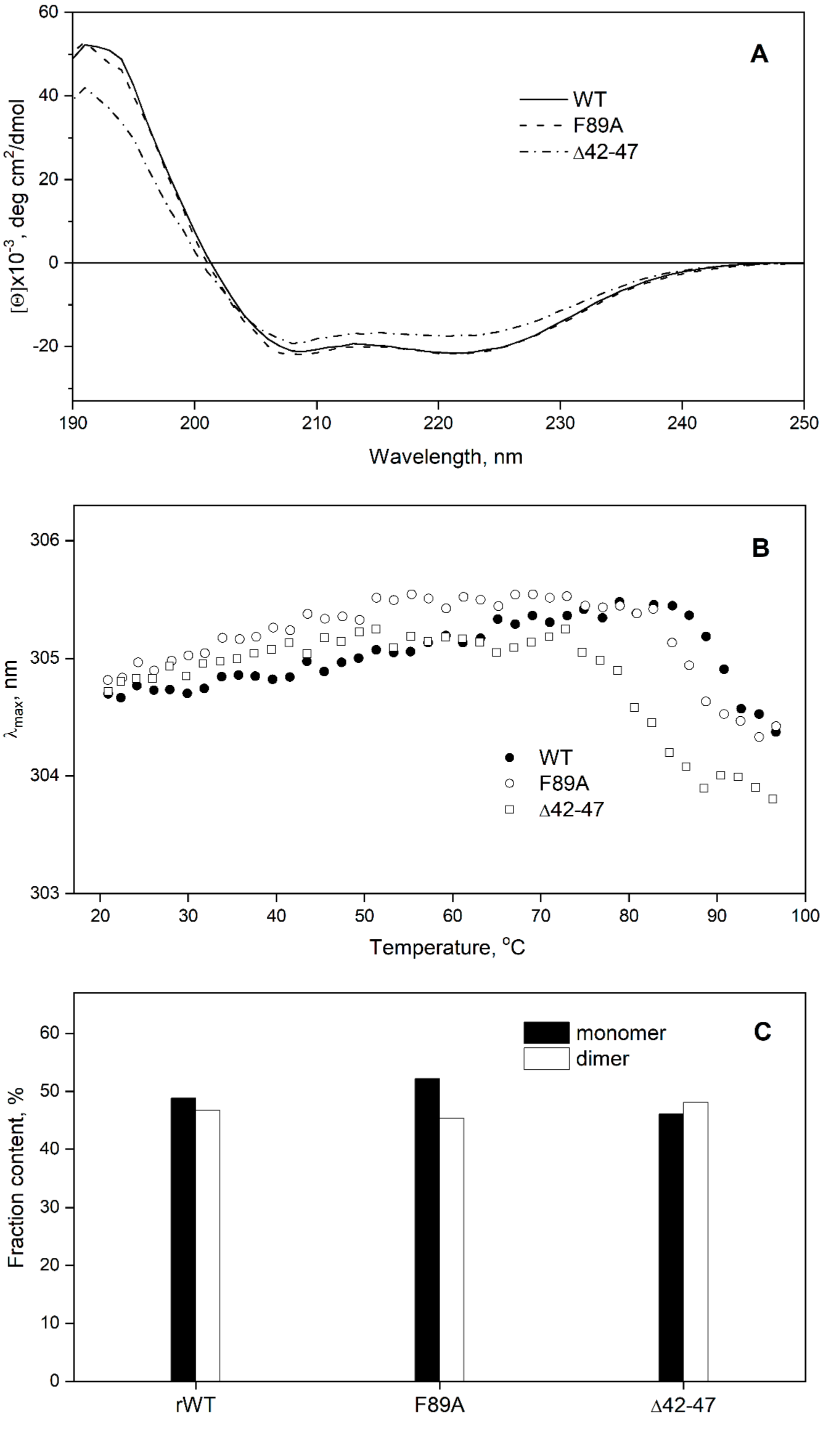

Figure S1. Comparison of structural properties of apo-forms of WT S100P and its F89A, Δ42-47 mutants, using far-UV CD spectroscopy at 20ºC (panel **A**), intrinsic fluorescence (**B**) and chemical crosslinking at 20 °C by 0.02% glutaraldehyde for 16 h (**C**) (10 mM

tricine-KOH, 1 mM EDTA-KOH, pH 7.4). Protein concentration was 10 μM (**A**, **B**) or 67 μM (**C**). Calcium depletion was performed according to ref. [113].

References

1. Kazakov, A.S.; Sokolov, A.S.; Permyakova, M.E.; Litus, E.A.; Uversky, V.N.; Permyakov, E.A.; Permyakov, S.E. Specific cytokines of interleukin-6 family interact with S100 proteins. *Cell Calcium* **2022**, *101*, 102520, doi:10.1016/j.ceca.2021.102520.
2. Andreeva, A.; Kulesha, E.; Gough, J.; Murzin, A.G. The SCOP database in 2020: expanded classification of representative family and superfamily domains of known protein structures. *Nucleic Acids Res* **2020**, *48*, D376-D382, doi:10.1093/nar/gkz1064.
3. Papagoras, C.; Tsiami, S.; Chrysanthopoulou, A.; Mitroulis, I.; Baraliakos, X. Serum granulocyte-macrophage colony-stimulating factor (GM-CSF) is increased in patients with active radiographic axial spondyloarthritis and persists despite anti-TNF treatment. *Arthritis Research & Therapy* **2022**, *24*, 195, doi:10.1186/s13075-022-02888-6.
4. Riccio, A.; De Caterina, M.; Natale, D.; Grimaldi, E.; Pronesti, G.; Montagnani, S.; Postiglione, L. Serum Levels of Granulocyte Macrophage Colony Stimulating Factor (GM-CSF) in a Group of Patients with Systemic Sclerosis. *International Journal of Immunopathology and Pharmacology* **1996**, *9*, 9-12, doi:10.1177/039463209600900102.
5. Balleari, E.; Bason, C.; Visani, G.; Gobbi, M.; Ottaviani, E.; Ghio, R. Serum levels of granulocyte-macrophage colony-stimulating factor and granulocyte colony-stimulating factor in treated patients with chronic myelogenous leukemia in chronic phase. *Haematologica* **1994**, *79*, 7-12.
6. Fiehn, C.; Wermann, M.; Pezzutto, A.; Hufner, M.; Heilig, B. [Plasma GM-CSF concentrations in rheumatoid arthritis, systemic lupus erythematosus and spondyloarthropathy]. *Z Rheumatol* **1992**, *51*, 121-126.
7. Carrieri, P.B.; Provitera, V.; De Rosa, T.; Tartaglia, G.; Gorga, F.; Perrella, O. Profile of cerebrospinal fluid and serum cytokines in patients with relapsing-remitting multiple sclerosis: a correlation with clinical activity. *Immunopharmacol Immunotoxicol* **1998**, *20*, 373-382, doi:10.3109/08923979809034820.
8. Kleiner, G.; Marcuzzi, A.; Zanin, V.; Monasta, L.; Zauli, G. Cytokine levels in the serum of healthy subjects. *Mediators Inflamm* **2013**, *2013*, 434010, doi:10.1155/2013/434010.
9. Kutukculer, N.; Vergin, C.; Cetingul, N.; Kavakli, K.; Caglayan, S.; Oztop, S.; Nisli, G. Plasma interleukin-3 (IL-3) and IL-7 concentrations in children with homozygous beta-thalassemia. *J Trop Pediatr* **1997**, *43*, 366-367, doi:10.1093/tropej/43.6.366.
10. Biancotto, A.; Wank, A.; Perl, S.; Cook, W.; Olnes, M.J.; Dagur, P.K.; Fuchs, J.C.; Langweiler, M.; Wang, E.; McCoy, J.P. Baseline levels and temporal stability of 27 multiplexed serum cytokine concentrations in healthy subjects. *Plos One* **2013**, *8*, e76091, doi:10.1371/journal.pone.0076091.

11. Xiu, M.H.; Lin, C.G.; Tian, L.; Tan, Y.L.; Chen, J.; Chen, S.; Tan, S.P.; Wang, Z.R.; Yang, F.D.; Chen, D.C., et al. Increased IL-3 serum levels in chronic patients with schizophrenia: Associated with psychopathology. *Psychiatry Res* **2015**, *229*, 225-229, doi:10.1016/j.psychres.2015.07.029.

12. Lee, H.L.; Jang, J.W.; Lee, S.W.; Yoo, S.H.; Kwon, J.H.; Nam, S.W.; Bae, S.H.; Choi, J.Y.; Han, N.I.; Yoon, S.K. Inflammatory cytokines and change of Th1/Th2 balance as prognostic indicators for hepatocellular carcinoma in patients treated with transarterial chemoembolization. *Scientific Reports* **2019**, *9*, 3260, doi:10.1038/s41598-019-40078-8.

13. Lee, Y.C.; Lee, K.H.; Lee, H.B.; Rhee, Y.K. Serum levels of interleukins (IL)-4, IL-5, IL-13, and interferon-gamma in acute asthma. *J Asthma* **2001**, *38*, 665-671, doi:10.1081/jas-100107544.

14. Joseph, J.; Benedict, S.; Safa, W.; Joseph, M. Serum interleukin-5 levels are elevated in mild and moderate persistent asthma irrespective of regular inhaled glucocorticoid therapy. *BMC Pulm Med* **2004**, *4*, 2, doi:10.1186/1471-2466-4-2.

15. Dimitrova, D.; Youroukova, V.; Ivanova-Todorova, E.; Tumangelova-Yuzeir, K.; Velikova, T. Serum levels of IL-5, IL-6, IL-8, IL-13 and IL-17A in pre-defined groups of adult patients with moderate and severe bronchial asthma. *Respir Med* **2019**, *154*, 144-154, doi:10.1016/j.rmed.2019.06.024.

16. Perret, J.; McDonald, C.; Apostolopoulos, V. Elevated serum interleukin-5 levels in severe chronic obstructive pulmonary disease. *Acta Biochim Biophys Sin (Shanghai)* **2017**, *49*, 560-563, doi:10.1093/abbs/gmx030.

17. Fischer, M.; Bijman, M.; Molin, D.; Cormont, F.; Uyttenhove, C.; van Snick, J.; Sundstrom, C.; Enblad, G.; Nilsson, G. Increased serum levels of interleukin-9 correlate to negative prognostic factors in Hodgkin's lymphoma. *Leukemia* **2003**, *17*, 2513-2516, doi:10.1038/sj.leu.2403123.

18. Dantas, A.T.; Marques, C.D.L.; da Rocha Junior, L.F.; Cavalcanti, M.B.; Gonçalves, S.M.C.; Cardoso, P.R.G.; Mariz, H.d.A.; Rego, M.J.B.d.M.; Duarte, A.L.B.P.; Pitta, I.d.R., et al. Increased Serum Interleukin-9 Levels in Rheumatoid Arthritis and Systemic Lupus Erythematosus: Pathogenic Role or Just an Epiphenomenon? *Disease Markers* **2015**, *2015*, 519638, doi:10.1155/2015/519638.

19. Huang, Y.; Cao, Y.; Zhang, S.; Gao, F. Association between low expression levels of interleukin-9 and colon cancer progression. *Exp Ther Med* **2015**, *10*, 942-946, doi:10.3892/etm.2015.2588.

20. Defendenti, C.; Sarzi-Puttini, P.; Saibeni, S.; Bollani, S.; Bruno, S.; Almasio, P.L.; Declich, P.; Atzeni, F. Significance of serum Il-9 levels in inflammatory bowel disease. *Int J Immunopathol Pharmacol* **2015**, *28*, 569-575, doi:10.1177/0394632015600535.

21. Lv, X.; Feng, L.; Ge, X.; Lu, K.; Wang, X. Interleukin-9 promotes cell survival and drug resistance in diffuse large B-cell lymphoma. *J Exp Clin Cancer Res* **2016**, *35*, 106, doi:10.1186/s13046-016-0374-3.

22. Ciprandi, G.; De Amici, M.; Castellazzi, A.M.; Tosca, M.A.; Marseglia, G. Serum IL-9 levels depend on allergen exposure: preliminary study. *Int Arch Allergy Immunol* **2011**, *154*, 246-248, doi:10.1159/000321111.

23. Yanaba, K.; Yoshizaki, A.; Asano, Y.; Kadono, T.; Sato, S. Serum interleukin 9 levels are increased in patients with systemic sclerosis: association with lower frequency and severity of pulmonary fibrosis. *J Rheumatol* **2011**, *38*, 2193-2197, doi:10.3899/jrheum.110268.

24. Martínez-Reyes, C.P.; Gómez-Arauz, A.Y.; Torres-Castro, I.; Manjarrez-Reyna, A.N.; Palomera, L.F.; Olivos-García, A.; Mendoza-Tenorio, E.; Sánchez-Medina, G.A.; Islas-Andrade, S.; Melendez-Mier, G., et al. Serum Levels of Interleukin-13 Increase in Subjects with Insulin Resistance but Do Not Correlate with Markers of Low-Grade Systemic Inflammation. *Journal of Diabetes Research* **2018**, *2018*, 7209872, doi:10.1155/2018/7209872.

25. Nabavi, M.; Arshi, S.; Bahrami, A.; Aryan, Z.; Bemanian, M.H.; Esmaeilzadeh, H.; Jalali, F.; Pousti, S.B.; Rezaei, N. Increased level of interleukin-13, but not interleukin-4 and interferon-γ in chronic rhinosinusitis with nasal polyps. *Allergologia et Immunopathologia* **2014**, *42*, 465-471, doi:10.1016/j.aller.2013.06.007.

26. Cingu, A.K.; Turkcu, F.M.; Aktas, S.; Sahin, A.; Ayyildiz, O. Serum IL-4, IL-12, IL-13, IL-27, and IL-33 levels in active and inactive ocular Behcet's disease. *International Ophthalmology* **2020**, *40*, 3441-3451, doi:10.1007/s10792-020-01530-1.

27. Spadaro, A.; Rinaldi, T.; Riccieri, V.; Valesini, G.; Taccari, E. Interleukin 13 in synovial fluid and serum of patients with psoriatic arthritis. *Ann Rheum Dis* **2002**, *61*, 174-176, doi:10.1136/ard.61.2.174.

28. Versace, A.G.; Bitto, A.; Ioppolo, C.; Aragona, C.O.; La Rosa, D.; Roberts, W.N.; D'Angelo, T.; Cinquegrani, A.; Cirmi, S.; Irrera, N., et al. IL-13 and IL-33 Serum Levels Are Increased in Systemic Sclerosis Patients With Interstitial Lung Disease. *Front Med (Lausanne)* **2022**, *9*, 825567, doi:10.3389/fmed.2022.825567.

29. Mielnik, P.; Chwalinska-Sadowska, H.; Wiesik-Szewczyk, E.; Maslinski, W.; Olesinska, M. Serum concentration of interleukin 15, interleukin 2 receptor and TNF receptor in patients with polymyositis and dermatomyositis: correlation to disease activity. *Rheumatol Int* **2012**, *32*, 639-643, doi:10.1007/s00296-010-1692-y.

30. Kaibe, M.; Ohishi, M.; Ito, N.; Yuan, M.; Takagi, T.; Terai, M.; Tatara, Y.; Komai, N.; Rakugi, H.; Ogihara, T. Serum Interleukin-15 Concentration in Patients With Essential Hypertension*. *American Journal of Hypertension* **2005**, *18*, 1019-1025, doi:10.1016/j.amjhyper.2005.02.014.

31. Jang, G.-C.; Kim, H.-Y.; Ahn, S.-Y.; Kim, D.-S. Raised serum interleukin 15 levels in Kawasaki disease. *Annals of the Rheumatic Diseases* **2003**, *62*, 264-266, doi:10.1136/ard.62.3.264.

32. Mengus, C.; Le Magnen, C.; Trella, E.; Yousef, K.; Bubendorf, L.; Provenzano, M.; Bachmann, A.; Heberer, M.; Spagnoli, G.C.; Wyler, S. Elevated levels of circulating IL-7 and

IL-15 in patients with early stage prostate cancer. *Journal of Translational Medicine* **2011**, *9*, 162, doi:10.1186/1479-5876-9-162.

33. Rentzos, M.; Cambouri, C.; Rombos, A.; Nikolaou, C.; Anagnostouli, M.; Tsoutsou, A.; Dimitrakopoulos, A.; Triantafyllou, N.; Vassilopoulos, D. IL-15 is elevated in serum and cerebrospinal fluid of patients with multiple sclerosis. *J Neurol Sci* **2006**, *241*, 25-29, doi:10.1016/j.jns.2005.10.003.
34. Fehniger, T.A.; Caligiuri, M.A. Interleukin 15: biology and relevance to human disease. *Blood* **2001**, *97*, 14-32, doi:10.1182/blood.v97.1.14.
35. Lauw, F.N.; Simpson, A.J.H.; Prins, J.M.; Smith, M.D.; Kurimoto, M.; van Deventer, S.J.H.; Speelman, P.; Chaowagul, W.; White, N.J.; van der Poll, T. Elevated Plasma Concentrations of Interferon (IFN)-γ and the IFN-γ—Inducing Cytokines Interleukin (IL)-18, IL-12, and IL-15 in Severe Melioidosis. *The Journal of Infectious Diseases* **1999**, *180*, 1878-1885, doi:10.1086/315155.
36. He, Z.; Jin, L.; Liu, Z.F.; Hu, L.; Dang, E.L.; Feng, Z.Z.; Li, Q.J.; Wang, G. Elevated serum levels of interleukin 21 are associated with disease severity in patients with psoriasis. *Br J Dermatol* **2012**, *167*, 191-193, doi:10.1111/j.1365-2133.2012.10899.x.
37. Pan, H.-F.; Wu, G.-C.; Fan, Y.-G.; Leng, R.-X.; Peng, H.; Zhou, M.; Li, B.-Z.; Zhu, Y.; Tao, J.-H.; Li, X.-P., et al. Decreased serum level of IL-21 in new-onset systemic lupus erythematosus patients. *Rheumatology International* **2013**, *33*, 2337-2342, doi:10.1007/s00296-013-2724-1.
38. Mizutani, H.; Tamagawa-Mineoka, R.; Nakamura, N.; Masuda, K.; Katoh, N. Serum IL-21 levels are elevated in atopic dermatitis patients with acute skin lesions. *Allergol Int* **2017**, *66*, 440-444, doi:10.1016/j.alit.2016.10.010.
39. Chen, H.M.; Liu, H.L.; Yang, Y.C.; Cheng, X.L.; Wang, Y.F.; Xing, F.F.; Zhao, Y.R. Serum IL-21 levels associated with chronic hepatitis B and hepatitis B-related liver failure. *Exp Ther Med* **2014**, *7*, 1013-1019, doi:10.3892/etm.2014.1533.
40. Ding, R.; Gao, W.; He, Z.; Liao, M.; Wu, F.; Zou, S.; Ma, L.; Liang, C.; Wu, Z. Effect of serum interleukin 21 on the development of coronary artery disease. *APMIS* **2014**, *122*, 842-847, doi:10.1111/apm.12246.
41. Temel, T.; Cansu, D.U.; Temel, H.E.; Ozakyol, A.H. Serum thrombopoietin levels and its relationship with thrombocytopenia in patients with cirrhosis. *Hepat Mon* **2014**, *14*, e18556, doi:10.5812/hepatmon.18556.
42. Usuki, K.; Tahara, T.; Iki, S.; Endo, M.; Osawa, M.; Kitazume, K.; Kato, T.; Miyazaki, H.; Urabe, A. Serum thrombopoietin level in various hematological diseases. *Stem Cells* **1996**, *14*, 558-565, doi:10.1002/stem.140558.
43. Ballmaier, M.; Schulze, H.; Strauβ, G.; Cherkaoui, K.; Wittner, N.; Lynen, S.; Wolters, S.; Bogenberger, J.; Welte, K. Thrombopoietin in Patients With Congenital Thrombocytopenia and Absent Radii: Elevated Serum Levels, Normal Receptor Expression, But Defective Reactivity to Thrombopoietin. *Blood* **1997**, *90*, 612-619, doi:10.1182/blood.V90.2.612.

44. Tsukishiro, S.; Suzumori, N.; Nishikawa, H.; Arakawa, A.; Suzumori, K. Preoperative serum thrombopoietin levels are higher in patients with ovarian cancer than with benign cysts. *European Journal of Obstetrics and Gynecology and Reproductive Biology* **2008**, *140*, 67-70, doi:10.1016/j.ejogrb.2005.10.037.

45. Makar, R.S.; Zhukov, O.S.; Sahud, M.A.; Kuter, D.J. Thrombopoietin levels in patients with disorders of platelet production: diagnostic potential and utility in predicting response to TPO receptor agonists. *Am J Hematol* **2013**, *88*, 1041-1044, doi:10.1002/ajh.23562.

46. Ezzat, M.H.; Hasan, Z.E.; Shaheen, K.Y. Serum measurement of interleukin-31 (IL-31) in paediatric atopic dermatitis: elevated levels correlate with severity scoring. *J Eur Acad Dermatol Venereol* **2011**, *25*, 334-339, doi:10.1111/j.1468-3083.2010.03794.x.

47. Ginaldi, L.; De Martinis, M.; Ciccarelli, F.; Saitta, S.; Imbesi, S.; Mannucci, C.; Gangemi, S. Increased levels of interleukin 31 (IL-31) in osteoporosis. *BMC Immunology* **2015**, *16*, 60, doi:10.1186/s12865-015-0125-9.

48. Rosine, N.; Etcheto, A.; Hendel-Chavez, H.; Seror, R.; Briot, K.; Molto, A.; Chanson, P.; Taoufik, Y.; Wendling, D.; Lories, R., et al. Increase In Il-31 Serum Levels Is Associated With Reduced Structural Damage In Early Axial Spondyloarthritis. *Scientific Reports* **2018**, *8*, 7731, doi:10.1038/s41598-018-25722-z.

49. Swierczynska, K.; Krajewski, P.K.; Nowicka-Suszko, D.; Bialynicki-Birula, R.; Krajewska, M.; Szepietowski, J.C. The Serum Level of IL-31 in Patients with Chronic Kidney Disease-Associated Pruritus: What Can We Expect? *Toxins (Basel)* **2022**, *14*, doi:10.3390/toxins14030197.

50. Raap, U.; Wichmann, K.; Bruder, M.; Stander, S.; Wedi, B.; Kapp, A.; Werfel, T. Correlation of IL-31 serum levels with severity of atopic dermatitis. *J Allergy Clin Immunol* **2008**, *122*, 421-423, doi:10.1016/j.jaci.2008.05.047.

51. Zeng, X.; Zhang, Z.; Gao, Q.Q.; Wang, Y.Y.; Yu, X.Z.; Zhou, B.; Xi, M.R. Clinical Significance of Serum Interleukin-31 and Interleukin-33 Levels in Patients of Endometrial Cancer: A Case Control Study. *Dis Markers* **2016**, *2016*, 9262919, doi:10.1155/2016/9262919.

52. Hassan, W.A.; Hamaad, G.A.; Sayed, E.A.; El Behisy, M.M.; Gomaa, M.K. Clinical significance of interleukin 27 serum concentration in patients with systemic sclerosis: relation to clinical, laboratory and radiological parameters. *Egyptian Rheumatology and Rehabilitation* **2019**, *46*, 101-107, doi:10.4103/err.err_63_18.

53. Swaminathan, S.; Hu, Z.; Rupert, A.W.; Higgins, J.M.; Dewar, R.L.; Stevens, R.; Chen, Q.; Rehm, C.A.; Metcalf, J.A.; Baseler, M.W., et al. Plasma Interleukin-27 (IL-27) Levels Are Not Modulated in Patients with Chronic HIV-1 Infection. *Plos One* **2014**, *9*, e98989, doi:10.1371/journal.pone.0098989.

54. Hassan, T.; Abdel Rahman, D.; Raafat, N.; Fathy, M.; Shehab, M.; Hosny, A.; Fawzy, R.; Zakaria, M. Contribution of interleukin 27 serum level to pathogenesis and prognosis in children with immune thrombocytopenia. *Medicine (Baltimore)* **2022**, *101*, e29504, doi:10.1097/MD.0000000000029504.

55. Jafarzadeh, A.; Nemati, M.; Rezayati, M.T. Serum levels of interleukin (IL)-27 in patients with ischemic heart disease. *Cytokine* **2011**, *56*, 153-156, doi:10.1016/j.cyto.2011.06.014.

56. Yan, A.; You, H.; Zhang, X. Levels of Interleukin 27 and Interleukin 35 in the Serum and Vitreous of Patients with Proliferative Diabetic Retinopathy. *Ocul Immunol Inflamm* **2018**, *26*, 273-279, doi:10.1080/09273948.2016.1203959.

57. Lukawska-Tatarczuk, M.; Franek, E.; Czupryniak, L.; Joniec-Maciejak, I.; Pawlak, A.; Wojnar, E.; Zielinski, J.; Mirowska-Guzel, D.; Mrozikiewicz-Rakowska, B. Sirtuin 1, Visfatin and IL-27 Serum Levels of Type 1 Diabetic Females in Relation to Cardiovascular Parameters and Autoimmune Thyroid Disease. *Biomolecules* **2021**, *11*, doi:1110 [pii]

10.3390/biom11081110.

58. Forrester, M.A.; Robertson, L.; Bayoumi, N.; Keavney, B.D.; Barker, R.N.; Vickers, M.A. Human interleukin-27: wide individual variation in plasma levels and complex inter-relationships with interleukin-17A. *Clin Exp Immunol* **2014**, *178*, 373-383, doi:10.1111/cei.12408.

59. Batebi, A.; Namavar-Jahromi, B.; Ali-Hassanzadeh, M.; Ahmadi, M.; Hosseini, M.S.; Gharesi-Fard, B. Evaluation of IL-17 and IL-35 Serum Levels in Patients with Preeclampsia. *J Reprod Infertil* **2019**, *20*, 237-243.

60. Gu, J.H.; Wang, X.G.; Wang, L.Q.; Zhou, L.N.; Tang, M.; Li, P.; Wu, X.Y.; Chen, M.B.; Zhang, Y. Serum level of interleukin-35 as a potential prognostic factor for gastric cancer. *Asia Pac J Clin Oncol* **2021**, *17*, 52-59, doi:10.1111/ajco.13403.

61. Chatrabnous, N.; Ghaderi, A.; Ariafar, A.; Razeghinia, M.S.; Nemati, M.; Jafarzadeh, A. Serum concentration of interleukin-35 and its association with tumor stages and FOXP3 gene polymorphism in patients with prostate cancer. *Cytokine* **2019**, *113*, 221-227, doi:10.1016/j.cyto.2018.07.006.

62. Li, Y.; Yao, L.; Liu, S.; JishengWu; Xia, L.; Shen, H.; Lu, J. Correlation between Serum IL-35 Levels and Bone Loss in Postmenopausal Women with Rheumatoid Arthritis. *Mediators Inflamm* **2019**, *2019*, 9139145, doi:10.1155/2019/9139145.

63. Eslami, M.; Rafiei, A.; Baghbanian, S.M.; Fattahi, S.; Yazdani, Z.; Valadan, R.; Kardan, M. Serum levels and genetic variation of IL-35 are associated with multiple sclerosis: a population-based case-control study. *Immunol Res* **2022**, *70*, 75-85, doi:10.1007/s12026-021-09246-9.

64. Tomcik, M.; Zerr, P.; Palumbo-Zerr, K.; Storkanova, H.; Hulejova, H.; Spiritovic, M.; Kodet, O.; Stork, J.; Becvar, R.; Vencovsky, J., et al. Interleukin-35 is upregulated in systemic sclerosis and its serum levels are associated with early disease. *Rheumatology (Oxford)* **2015**, *54*, 2273-2282, doi:10.1093/rheumatology/kev260.

65. Dantas, A.T.; Goncalves, S.M.; Pereira, M.C.; Goncalves, R.S.; Marques, C.D.; Rego, M.J.; Pitta Ida, R.; Duarte, A.L.; Pitta, M.G. Increased IL-35 serum levels in systemic sclerosis and association with pulmonary interstitial involvement. *Clin Rheumatol* **2015**, *34*, 1621-1625, doi:10.1007/s10067-015-3006-y.

66. Kawakami, M.; Tsutsumi, H.; Kumakawa, T.; Abe, H.; Hirai, M.; Kurosawa, S.; Mori, M.; Fukushima, M. Levels of serum granulocyte colony-stimulating factor in patients with infections. *Blood* **1990**, *76*, 1962-1964, doi:S0006-4971(20)82103-X [pii].

67. Bordbar, E.; Malekzadeh, M.; Ardekani, M.T.; Doroudchi, M.; Ghaderi, A. Serum levels of G-CSF and IL-7 in Iranian breast cancer patients. *Asian Pac J Cancer Prev* **2012**, *13*, 5307-5312, doi:10.7314/apjcp.2012.13.10.5307.

68. Pauksen, K.; Elfman, L.; Ulfgren, A.K.; Venge, P. Serum levels of granulocyte-colony stimulating factor (G-CSF) in bacterial and viral infections, and in atypical pneumonia. *Br J Haematol* **1994**, *88*, 256-260, doi:10.1111/j.1365-2141.1994.tb05015.x.

69. Morozumi, K.; Namiki, S.; Kudo, T.; Aizawa, M.; Ioritani, N. Serum G-CSF May Be a More Valuable Biomarker than Image Evaluation in G-CSF-Producing Urothelial Carcinoma: A Case Report. *Case Rep Oncol* **2017**, *10*, 377-382, doi:10.1159/000472250.

70. Watari, K.; Asano, S.; Shirafuji, N.; Kodo, H.; Ozawa, K.; Takaku, F.; Kamachi, S. Serum granulocyte colony-stimulating factor levels in healthy volunteers and patients with various disorders as estimated by enzyme immunoassay. *Blood* **1989**, *73*, 117-122.

71. Gross-Weege, W.; Dumon, K.; Dahmen, A.; Schneider, E.M.; Röher, H.D. Granulocyte colony-stimulating factor (G-CSF) serum levels in surgical intensive care patients. *Infection* **1997**, *25*, 213-216, doi:10.1007/bf01713146.

72. Triantafillidis, J.K.; Merikas, E.; Govosdis, V.; Konstandellou, E.; Cheracakis, P.; Barbatzas, C.; Tzourmakliotis, D.; Peros, G. Increased fasting serum levels of growth hormone and gastrin in patients with gastric and large bowel cancer. *Hepatogastroenterology* **2003**, *50 Suppl 2*, cclvi-cclx.

73. Elkarow, M.H.; Hamdy, A. A Suggested Role of Human Growth Hormone in Control of the COVID-19 Pandemic. *Front Endocrinol (Lausanne)* **2020**, *11*, 569633, doi:10.3389/fendo.2020.569633.

74. Akirov, A.; Masri-Iraqi, H.; Dotan, I.; Shimon, I. The Biochemical Diagnosis of Acromegaly. *J Clin Med* **2021**, *10*, doi:10.3390/jcm10051147.

75. Ishikawa, M.; Yokoya, S.; Tachibana, K.; Hasegawa, Y.; Yasuda, T.; Tokuhiro, E.; Hashimoto, Y.; Tanaka, T. Serum levels of 20-kilodalton human growth hormone (GH) are parallel those of 22-kilodalton human GH in normal and short children. *J Clin Endocrinol Metab* **1999**, *84*, 98-104, doi:10.1210/jcem.84.1.5402.

76. Christiansen, M. Placental growth hormone and growth hormone binding protein are first trimester maternal serum markers of Down syndrome. *Prenat Diagn* **2009**, *29*, 1249-1255, doi:10.1002/pd.2398.

77. Mittal, P.; Espinoza, J.; Hassan, S.; Kusanovic, J.P.; Edwin, S.S.; Nien, J.K.; Gotsch, F.; Than, N.G.; Erez, O.; Mazaki-Tovi, S., et al. Placental growth hormone is increased in the maternal and fetal serum of patients with preeclampsia. *J Matern Fetal Neonatal Med* **2007**, *20*, 651-659, doi:10.1080/14767050701463571.

78. Juhasz, E.; Kiss, E.; Simonova, E.; Patocs, A.; Reismann, P. Serum prolactin as a biomarker for the study of intracerebral dopamine effect in adult patients with phenylketonuria: a cross-sectional monocentric study. *Eur J Med Res* **2016**, *21*, 22, doi:10.1186/s40001-016-0212-2.

79. Jacobi, A.M.; Rohde, W.; Ventz, M.; Riemekasten, G.; Burmester, G.R.; Hiepe, F. Enhanced serum prolactin (PRL) in patients with systemic lupus erythematosus: PRL levels are related to the disease activity. *Lupus* **2001**, *10*, 554-561, doi:10.1191/096120301701549688.

80. Keen, M.A.; Hassan, I. Serum prolactin levels in psoriasis and its association with disease activity: a case-control study. *Indian J Dermatol* **2014**, *59*, 562-566, doi:10.4103/0019-5154.143512.

81. Cohen, A.D.; Cohen, Y.; Maislos, M.; Buskila, D. Prolactin serum level in patients with breast cancer. *Isr Med Assoc J* **2000**, *2*, 287-289.

82. Al-Nami, M.S.; Al-Kuraishy, H.M.; Al-Gareeb, A.I.; Al-Mamoori, F. Metabolic profile and prolactin serum levels in men with type 2 diabetes mellitus: Old-new rubric. *Int J Crit Illn Inj Sci* **2019**, *9*, 120-126, doi:10.4103/IJCIIS.IJCIIS_40_19.

83. Szczepankiewicz, D.; Sobkowiak, P.; Narozna, B.; Wojsyk-Banaszak, I.; Breborowicz, A.; Szczepankiewicz, A. Leptin gene polymorphism affects leptin level in childhood asthma. *World J Pediatr* **2018**, *14*, 601-606, doi:10.1007/s12519-018-0182-2.

84. Kvistad, S.S.; Myhr, K.M.; Holmoy, T.; Benth, J.S.; Wergeland, S.; Beiske, A.G.; Bjerve, K.S.; Hovdal, H.; Midgard, R.; Sagen, J.V., et al. Serum levels of leptin and adiponectin are not associated with disease activity or treatment response in multiple sclerosis. *J Neuroimmunol* **2018**, *323*, 73-77, doi:10.1016/j.jneuroim.2018.07.011.

85. Fahmi, R.M.; Kamel, A.E.; Elsayed, D.A.; Zidan, A.A.; Sarhan, N.T. Serum levels of leptin and adiponectin in patients with multiple sclerosis. *The Egyptian Journal of Neurology, Psychiatry and Neurosurgery* **2021**, *57*, 114, doi:10.1186/s41983-021-00369-2.

86. Demiray, G.; Degirmencioglu, S.; Ugurlu, E.; Yaren, A. Effects of Serum Leptin and Resistin Levels on Cancer Cachexia in Patients With Advanced-Stage Non-Small Cell Lung Cancer. *Clin Med Insights Oncol* **2017**, *11*, 1179554917690144, doi:10.1177/1179554917690144.

87. Duan, D.M.; Jhang, J.Y.; Wu, S.; Teng, M.S.; Hsu, L.A.; Ko, Y.L. Modification effect of sex and obesity on the correlation of LEP polymorphisms with leptin levels in Taiwanese obese women. *Mol Genet Genomic Med* **2020**, *8*, e1113, doi:10.1002/mgg3.1113.

88. Ko, B.J.; Lee, M.; Park, H.S.; Han, K.; Cho, G.J.; Hwang, T.G.; Kim, J.H.; Lee, S.H.; Lee, H.Y.; Kim, S.M. Elevated vaspin and leptin levels are associated with obesity in prepubertal Korean children. *Endocr J* **2013**, *60*, 609-616, doi:10.1507/endocrj.ej12-0384.

89. Teresa Gonzalez-Garza, M.; Elva Cruz-Vega, D.; Maldonado-Bernal, C. IL10 as Cancer Biomarker. **2021**, 10.5772/intechopen.90806, doi:10.5772/intechopen.90806.

90. Sobhan, M.R.; Farshchian, M.; Hoseinzadeh, A.; Ghasemibasir, H.R.; Solgi, G. Serum Levels of IL-10 and IL-22 Cytokines in Patients with Psoriasis. *Iran J Immunol* **2016**, *13*, 317-323.

91. Hussien, D.T.; Shabana, A.A.; Hassan, A.S.; Elmarghany, E.B. Assessment of serum interleukin-20 level in rheumatoid arthritis patients: Relation to disease activity and ultrasound measures. *The Egyptian Rheumatologist* **2022**, *44*, 181-186, doi:10.1016/j.ejr.2022.01.002.

92. Michalak-Stoma, A.; Bartosinska, J.; Kowal, M.; Juszkiewicz-Borowiec, M.; Gerkowicz, A.; Chodorowska, G. Serum levels of selected Th17 and Th22 cytokines in psoriatic patients. *Dis Markers* **2013**, *35*, 625-631, doi:10.1155/2013/856056.

93. Naumnik, W.; Naumnik, B.; Niklińska, W.; Ossolińska, M.; Chyczewska, E. Clinical Implications of Hepatocyte Growth Factor, Interleukin-20, and Interleukin-22 in Serum and Bronchoalveolar Fluid of Patients with Non-Small Cell Lung Cancer. In *Advancements in Clinical Research*, Pokorski, M., Ed. Springer International Publishing: Cham, 2016; 10.1007/5584_2016_66pp. 41-49.

94. Kragstrup, T.W.; Otkjaer, K.; Holm, C.; Jorgensen, A.; Hokland, M.; Iversen, L.; Deleuran, B. The expression of IL-20 and IL-24 and their shared receptors are increased in rheumatoid arthritis and spondyloarthropathy. *Cytokine* **2008**, *41*, 16-23, doi:10.1016/j.cyto.2007.10.004.

95. Valentina, M.; Jan, F.; Peder, N.L.; Bo, Z.; Hongjie, D.; Pernille, K. Cytokine detection and simultaneous assessment of rheumatoid factor interference in human serum and synovial fluid using high-sensitivity protein arrays on plasmonic gold chips. *BMC Biotechnol* **2015**, *15*, 73, doi:10.1186/s12896-015-0186-0.

96. Aydogdu, E.; Pamuk, O.N.; Donmez, S.; Pamuk, G.E. Decreased interleukin-20 level in patients with systemic sclerosis: are they related with angiogenesis? *Clin Rheumatol* **2013**, *32*, 1599-1603, doi:10.1007/s10067-013-2317-0.

97. Wang, T.; Zhang, Z.; Xing, H.; Wang, L.; Zhang, G.; Yu, N.; Wang, J.; Guo, W.; Jiang, J. Elevated Th22 cells and related cytokines in patients with epithelial ovarian cancer. *Medicine (Baltimore)* **2017**, *96*, e8359, doi:10.1097/MD.0000000000008359.

98. Tsirakis, G.; Pappa, C.A.; Kolovou, A.; Kokonozaki, M.; Neonakis, I.; Alexandrakis, M.G. Clinical significance of interleukin-22 in multiple myeloma. *Hematology* **2015**, *20*, 143-147, doi:10.1179/1607845414Y.0000000182.

99. Souza, J.M.; Matias, B.F.; Rodrigues, C.M.; Murta, E.F.; Michelin, M.A. IL-17 and IL-22 serum cytokine levels in patients with squamous intraepithelial lesion and invasive cervical carcinoma. *Eur J Gynaecol Oncol* **2013**, *34*, 466-468.

100. Abdullah, H.N.; Abdulwahid, A.G. Expression of Serum IL-22, IL-23, and TLR9 as Tumor Markers in Untreated Breast Cancer Patients. *Journal of drug delivery* **2020**, *10*, 472-476.

101. Thomas-Dupont, P.; Remes-Troche, J.M.; Izaguirre-Hernández, I.Y.; Sánchez-Vargas, L.A.; Maldonado-Rentería, M.d.J.; Hernández-Flores, K.G.; Torre, A.; Bravo-Sarmiento, E.; Vivanco-Cid, H. Elevated circulating levels of IL-21 and IL-22 define a cytokine signature profile in type 2 autoimmune hepatitis patients. *Annals of Hepatology* **2016**, *15*, 550-558, doi:10.5604/16652681.1203152.

102. Khoshroo, M.; Yazdanpanah, M.J.; Yasrebi, S. Serum Interleukin-24 Levels in Gastric and Breast Cancers and Non-cancerous Inflammations. *Middle East Journal of Cancer* **2021**, *12*, 183-189, doi:10.30476/mejc.2020.82945.1122.

103. Li, R.C.; Guo, J.; Su, L.C.; Huang, A.F. Elevated levels of IL-24 in systemic lupus erythematosus patients. *Lupus* **2019**, *28*, 748-754, doi:10.1177/0961203319845476.

104. Brilland, B.; Bach-Bunner, M.; Gomes, C.N.; Larochette, V.; Foucher, E.; Plaisance, M.; Saulnier, P.; Costedoat-Chalumeau, N.; Ghillani, P.; Belizna, C., et al. Serum Interleukin-26 Is a New Biomarker for Disease Activity Assessment in Systemic Lupus Erythematosus. *Front Immunol* **2021**, *12*, 663192, doi:10.3389/fimmu.2021.663192.

105. Shen, Y.; Dong, X.; Liu, J.; Lv, H.; Ge, Y. Serum Interleukin-26 is a Potential Biomarker for the Differential Diagnosis of Neurosyphilis and Syphilis at Other Stages. *Infect Drug Resist* **2022**, *15*, 3693-3702, doi:10.2147/IDR.S366308.

106. Louhaichi, S.; Mlika, M.; Hamdi, B.; Hamzaoui, K.; Hamzaoui, A. Sputum IL-26 Is Overexpressed in Severe Asthma and Induces Proinflammatory Cytokine Production and Th17 Cell Generation: A Case-Control Study of Women. *J Asthma Allergy* **2020**, *13*, 95-107, doi:10.2147/JAA.S229522.

107. Kaabachi, W.; Bouali, E.; Berraïes, A.; Dhifallh, I.B.; Hamdi, B.; Hamzaoui, K.; Hamzaoui, A. Interleukin-26 is overexpressed in Behçet's disease and enhances Th17 related −cytokines. *Immunology Letters* **2017**, *190*, 177-184, doi:10.1016/j.imlet.2017.08.008.

108. Corvaisier, M.; Delneste, Y.; Jeanvoine, H.; Preisser, L.; Blanchard, S.; Garo, E.; Hoppe, E.; Barre, B.; Audran, M.; Bouvard, B., et al. IL-26 is overexpressed in rheumatoid arthritis and induces proinflammatory cytokine production and Th17 cell generation. *PLoS Biol* **2012**, *10*, e1001395, doi:10.1371/journal.pbio.1001395.

109. Liao, A.P.; Salajegheh, M.; Nazareno, R.; Kagan, J.C.; Jubin, R.G.; Greenberg, S.A. Interferon beta is associated with type 1 interferon-inducible gene expression in dermatomyositis. *Ann Rheum Dis* **2011**, *70*, 831-836, doi:10.1136/ard.2010.139949.

110. Berman, H.M.; Westbrook, J.; Feng, Z.; Gilliland, G.; Bhat, T.N.; Weissig, H.; Shindyalov, I.N.; Bourne, P.E. The Protein Data Bank. *Nucleic Acids Res.* **2000**, *28*, 235-242.

111. Jumper, J.; Evans, R.; Pritzel, A.; Green, T.; Figurnov, M.; Ronneberger, O.; Tunyasuvunakool, K.; Bates, R.; Zidek, A.; Potapenko, A., et al. Highly accurate protein structure prediction with AlphaFold. *Nature* **2021**, *596*, 583-589, doi:10.1038/s41586-021-03819-2.

112. Desta, I.T.; Porter, K.A.; Xia, B.; Kozakov, D.; Vajda, S. Performance and Its Limits in Rigid Body Protein-Protein Docking. *Structure* **2020**, *28*, 1071-1081 e1073, doi:10.1016/j.str.2020.06.006.

113. Blum, H.E.; Lehky, P.; Kohler, L.; Stein, E.A.; Fischer, E.H. Comparative properties of vertebrate parvalbumins. *J.Biol.Chem.* **1977**, *252*, 2834-2838.
